# Non-Syndromic Cleft Lip with or without Cleft Palate: Genome-Wide Association Study in Europeans Identifies a Suggestive Risk Locus at 16p12.1 and Supports *SH3PXD2A* as a Clefting Susceptibility Gene

**DOI:** 10.3390/genes10121023

**Published:** 2019-12-07

**Authors:** Iris ALM van Rooij, Kerstin U Ludwig, Julia Welzenbach, Nina Ishorst, Michelle Thonissen, Tessel E Galesloot, Edwin Ongkosuwito, Stefaan J Bergé, Khalid Aldhorae, Augusto Rojas-Martinez, Lambertus ALM Kiemeney, Joris Robert Vermeesch, Han Brunner, Nel Roeleveld, Koen Devriendt, Titiaan Dormaar, Greet Hens, Michael Knapp, Carine Carels, Elisabeth Mangold

**Affiliations:** 1 Department for Health Evidence, Radboud Institute for Health Sciences, Radboud university medical center, 6500 HB Nijmegen, The Netherlands; iris.vanrooij@radboudumc.nl (I.A.v.R.); tessel.galesloot@radboudumc.nl (T.E.G.); bart.kiemeney@radboudumc.nl (L.A.K.); nel.roeleveld@radboudumc.nl (N.R.); 2 Institute of Human Genetics, University of Bonn, 53127 Bonn, Germany; kerstin.ludwig@uni-bonn.de (K.U.L.); jwel@uni-bonn.de (J.W.); Nina.Ishorst@uni-bonn.de (N.I.); 3 Department of Dentistry, Radboud Institute for Health Sciences, Section of Orthodontics and Craniofacial Biology, Radboud university medical center, 6500 HB Nijmegen, The Netherlands; michelle.thonissen@gmail.com (M.T.); Edwin.Ongkosuwito@radboudumc.nl (E.O.); 4 Department of Oral and Maxillofacial Surgery, Radboud university medical center, 6500 HB Nijmegen, The Netherlands; stefaan.berge@radboudumc.nl; 5 Orthodontic Department, College of Dentistry, Thamar University, Thamar, Yemen; drdurai2008@gmail.com; 6 Tecnologico de Monterrey, School of Medicine, and Universidad Autonoma de Nuevo Leon, Centro de Investigación y Desarrollo en Ciencias de la Salud, Monterrey 64460, Mexico; augusto.rojasmtz@tec.mx; 7 Department of Urology, Radboud Institute for Health Sciences, Radboud university medical center, 6500 HB Nijmegen, The Netherlands; 8 Department of Human Genetics, KU Leuven, 3000 Leuven, Belgiumcarine.carels@kuleuven.be (C.C.); 9 Department of Human Genetics, and Donders Institute for Brain, Cognition and Behaviour, Radboud university medical center, 6500 HB Nijmgen, The Netherlands; han.brunner@radboudumc.nl; 10 Department of Clinical Genetics, and GROW School for Oncology and Developmental Biology, Maastricht University Medical Center, 6202 AZ Maastricht, The Netherlands; 11 Center for Human Genetics, University Hospitals Leuven, KU Leuven, 3000 Leuven, Belgium; koenraad.devriendt@uzleuven.be; 12 Department of Imaging and Pathology, KU Leuven, 3000 Leuven, Belgium; titiaan.dormaar@uzleuven.be; 13 Oral and Maxillofacial Surgery, University Hospitals Leuven, 3000 Leuven, Belgium; 14 Department of Neurosciences, Experimental Otorhinolaryngology, KU Leuven, 3000 Leuven, Belgium; greet.hens@uzleuven.be; 15 Institute of Medical Biometry, Informatics and Epidemiology, University of Bonn, 53127 Bonn, Germany; knapp@imbie.meb.uni-bonn.de; 16 Orthodontics, University Hospitals KU Leuven, 3000 Leuven, Belgium

**Keywords:** congenital malformation, orofacial cleft, cleft lip with or without cleft palate, genome-wide association study

## Abstract

Non-syndromic cleft lip with or without cleft palate (nsCL/P) ranks among the most common human congenital malformations, and has a multifactorial background in which both exogenous and genetic risk factors act in concert. The present report describes a genome-wide association study (GWAS) involving a total of 285 nsCL/P patients and 1212 controls from the Netherlands and Belgium. Twenty of the 40 previously reported nsC/LP susceptibility loci were replicated, which underlined the validity of this sample. SNV-based analysis of the data identified an as yet unreported suggestive locus at chromosome 16p12.1 (*p*-value of the lead SNV: 4.17 × 10^−7^). This association was replicated in two of three patient/control replication series (Central European and Yemeni). Gene analysis of the GWAS data prioritized *SH3PXD2A* at chromosome 10q24.33 as a candidate gene for nsCL/P. To date, support for this gene as a cleft gene has been restricted to data from zebrafish and a knockout mouse model. The present GWAS was the first to implicate *SH3PXD2A* in non-syndromic cleft formation in humans. In summary, although performed in a relatively small sample, the present GWAS generated novel insights into nsCL/P etiology.

## 1. Introduction

Orofacial clefting represents the second most common congenital malformation in humans, after various forms of heart defects all combined [1]. Despite advances in surgical correction, the disorder has lifelong implications for the health and social integration of those affected. Improved understanding of cleft etiology may facilitate development of new preventative measures and therapeutic approaches, and may improve genetic counseling for families at risk.

Clefting can occur either as part of a complex malformation syndrome or as an isolated anomaly, and several cleft subphenotypes have been defined according to the affected anatomical structures. The most frequent subphenotype is non-syndromic cleft lip with or without cleft palate (nsCL/P). In European populations, the estimated prevalence of nsCL/P is around 1:1000 [1]. The etiology of nsCL/P is multifactorial, whereby genetic risk factors, environmental exposures, and potential gene–environment interactions all contribute to disease susceptibility. The estimated contribution of all combined genetic factors to nsCL/P is 90% [2]. 

Since 1989, diverse genetic approaches have been used to identify genes and pathways underlying nsCL/P, including linkage and candidate gene studies. However, prior to the commencement of the genomics era around a decade ago, extensive research efforts had identified only two common genetic factors that could be considered true nsCL/P-associated risk factors: (1) the regulatory region of the *Interferon Regulatory Factor 6* (*IRF6*), which was identified in a candidate gene association study; and (2) the *Forkhead Box E1* (*FOXE1*) risk locus, which was identified in a meta-analysis of linkage data [3,4]. In the genomics era, new DNA sequencing techniques have enabled whole-exome sequencing, which has led to the identification of potential nsCL/P susceptibility variants in *Cadherin 1* (*CDH1*) and a small number of other genes [5,6,7]. Variants detected to date via exome sequencing have been dominant, heterozygous, and can be important for the respective family as carriers of such variants can be at high risk. However, these are rare findings, and currently explain only a small fraction of patients.

Many key genetic findings in nsCL/P have resulted from another powerful tool of the genomics era, i.e., the genome-wide association study (GWAS) and follow-up analysis approach. To date, this approach has identified a total of 38 common risk loci for nsCL/P [8,9,10,11,12,13,14,15,16,17,18,19,20].

At the time of writing, a total 40 common nsCL/P risk loci are known. However, these account for only a modest proportion of the genetic variance of nsCL/P, e.g., up to 30% of the narrow-sense heritability in the European population [19]. Thus, the existence of further common risk loci must be assumed. Given the tremendous success of the nsCL/P GWAS and follow-up study approach over the past decade, further GWAS appear to be warranted.

The present report describes a GWAS in a medium-sized nsCL/P case/control sample of European ethnicity recruited in the Netherlands and Belgium. In addition to the SNV-wise evaluation of data, gene-based and pathway analyses were performed. In these analyses, genetic marker data were aggregated to the level of whole genes or biological processing to test the joint association of all markers in the gene with the phenotype of interest, thereby increasing statistical power. 

## 2. Materials and Methods 

### 2.1. Ethics Statement

The study was approved by the Regional Committee on Research Involving Human Subjects Arnhem-Nijmegen, and the Review Board for Clinical Studies of the University Hospital KU Leuven. All study procedures were performed in accordance with the principles of the Declaration of Helsinki. The NBS protocol was approved by the Regional Committee on Research Involving Human Subjects Arnhem-Nijmegen. Written informed consent was obtained from all patients/participants, or their parents/legal guardians in the case of legal minors.

### 2.2. GWAS Patients

Patients were part of the AGORA project (Aetiological Research into Genetic and Occupational/Environmental Risk Factors for Anomalies in Children) [21]. The AGORA project commenced in 2005 and has established a large data and biobank of DNA samples and clinical and questionnaire data from: (i) children with congenital malformations or childhood cancer, (ii) their respective parents, and (iii) controls. Patients are recruited at two sites: (1) the Radboud University Medical Center (Radboudumc) Nijmegen, The Netherlands; and (2) University Hospital KU, Leuven, Belgium. Only patients with nsCL/P were eligible for inclusion in the present study. Patients with other cleft phenotypes, such as an isolated cleft palate, and patients with a possible specific malformation syndrome, intellectual disability, or other anomalies were excluded from the present study. 

In the Nijmegen initiative, collection of data and biomaterials from patients with clefting commenced in 2007. Intraoperative blood or saliva samples were collected, and parents were asked to complete a questionnaire and to donate blood or saliva samples. The questionnaire addresses demographics and family history, among other factors. At the beginning of 2012, three “biomaterial donation days” were arranged to collect blood or saliva from clefting patients who had been treated at Radboudumc prior to 2007. At these sessions, blood or saliva samples and questionnaire data were also collected from parents.

In the Leuven initiative, collection of data and biomaterials from patients with clefting commenced in 2010. Blood samples and clinical and questionnaire data (e.g., demographics and family history) were collected from pediatric patients and their parents. 

At both Leuven and Nijmegen clinical sites, all pediatric clefting patients underwent a clinical examination by an orthodontist, a maxillofacial surgeon, a plastic surgeon, and a clinical geneticist from the Cleft Lip and Palate team. To determine phenotype classification in the present cohort, data were retrieved from the respective medical charts. 

The present analyses were performed using DNA samples from nsCL/P patients who were treated and followed up at: (1) Radboudumc (*n* = 219); or (2) University Hospital of KU Leuven (*n* = 66). The initial patient sample therefore comprised 285 nsCL/P patients. All patients were of self-reported European ancestry. Ancestral background in the Dutch and Belgian samples was assessed by identifying the origins of the grandparents and the parents, respectively.

### 2.3. GWAS Controls

Population-based controls (*n* = 1212) were drawn from the Nijmegen Biomedical Study (NBS), a population-based survey conducted by the Department for Health Evidence and the Department of Laboratory Medicine of the Radboud University Medical Center. This cohort was established to generate a universal reference population for the investigation of genetic variation and lifestyle and environmental exposures for a variety of traits and diseases in case/control studies [22]. A total of 22,451 age- and sex-stratified, randomly selected inhabitants of the municipality of Nijmegen received an invitation to fill out a postal questionnaire on lifestyle and medical history, and to donate an 8.5 mL blood sample in a serum separator tube and a 10 mL EDTA blood sample. The overall response to the questionnaire was 42% (*n* = 9350), and 69% (*n* = 6468) of the respondents donated blood samples. 

### 2.4. GWAS Genotyping and Quality Control (QC)

Within the AGORA project, standard methods are used to extract DNA from blood collected in EDTA tubes or saliva specimens collected in ORAgene containers (DNA Genotek Inc., Ottawa, Canada). In the present study, genotyping of the 285 nsCL/P patients from AGORA was performed using the Illumina OmniExpressExome Array (Illumina, San Diego, CA, USA). Within the NBS project, DNA was genotyped using Illumina chips. For the present analyses, Illumina’s OmniExpress Array (Illumina, San Diego, CA, USA) genotype data for the 1212 NBS participants were available. A total of 718,286 SNVs were present in both patients and controls. 

Exclusion criteria for patient and control data were: (i) any discrepancy between documented and genotyped sex; (ii) a call rate of <99%; or (iii) evidence from multidimensional scaling (MDS) of ethnic outlier status (Figure S1). Un-relatedness between individuals was evaluated using the program KING [23].

Markers were excluded from the analysis if the minor allele frequency (MAF) was <1% or the call rate was <95% in either patients or controls. In addition, markers were excluded if there was deviation from Hardy–Weinberg equilibrium at *p* < 10^−4^ in controls or *p* < 10^−6^ in patients. 

### 2.5. Imputation of GWAS Data

The combined post-QC dataset for patients and controls was subjected to imputation using the June 2014 release of the 1000 Genomes Project and the program IMPUTE2 [24]. SNVs with an INFO score ≥ 0.4 and a MAF > 1% were then tested for association using SNPTEST [25]. For the logistic regression analysis, the first five components obtained from MDS were used as covariates. 

### 2.6. Genome-Wide Association Analysis 

Four patients were excluded due to gender incompatibility (discrepancy between documented and genotyped sex). Two patients and one control sample were excluded due to a call rate < 99%. Five patients and 24 controls were excluded due to relatedness. Fifteen patients were excluded due to ethnic outlier status. Following these QC procedures, 259 patients and 1187 controls remained for further analysis. In total, 8,785,346 imputed SNVs with an INFO score ≥ 0.4 and a MAF > 1% were finally tested. For each SNV passing QC, a logistic regression model was considered, with the additively coded SNV as the predictor variable and the first five MDS coordinates as covariates. The *p*-value of the likelihood ratio test of no association was then calculated. 

### 2.7. Replication Analysis for Two Interesting SNVs

Two SNVs were of particular interest, as they had small *p*-values and were located in regions not reported to be nsCL/P risk loci. For these two interesting candidate SNVs (rs73145631 at chromosome 12, and rs56383345 at chromosome 16), replication was performed by genotyping these SNVs in three case/control nsCL/P samples: (i) 223 nsCL/P patients and 978 controls of Central European descent (sample presented in Mangold et al., 2010); (ii) 156 nsCL/P patients and 337 controls from the Chiapas, Mexico (sample presented in Rojas-Martinez et al. 2013); and (iii) 231 nsCL/P patients and 422 controls from Yemen (sample presented in Böhmer et al., 2014) [10,26,27].

A MassARRAY genotyping assay was designed using the Assay Design Suite Software v 1.0 Software (AGENA Bioscience, San Diego, USA). The genotyping assay contained these two SNVs of interest and 26 SNVs from other projects. SNVs were genotyped using the MassARRAY system and end-point PCR, followed by matrix-assisted laser desorption/ionization time-of-flight (MALDI-ToF) mass spectrometry (AGENA Bioscience, San Diego, USA). Data analysis was performed using the AGENA Spectrodesigner Software package. Individual genotypes were assigned using the AGENA Typer Analysis software. Primers were synthesized at Metabion, Germany (individual primer sequences available upon request). Intra- and interplate duplicates were included for quality control purposes. No genotype inconsistencies were observed. 

Association statistics were calculated by applying the Armitage-trend test separately for each sample cohort. For each SNV, relative risks of the three replication cohorts were combined using fixed-effect meta-analysis.

### 2.8. Prioritization of Candidate Genes

To prioritize candidate genes from the present GWAS dataset, SNV data with an INFO score > 0.6 were uploaded into FUMAGWAS (https://fuma.ctglab.nl/) [28]. Gene and gene-set analyses implemented in FUMAGWAS were based on GWAS summary statistics. In the present study, these analyses were performed with MAGMA, a tool that can be used for gene and gene-set analysis from GWAS data [29]. Input SNVs were mapped to 18,644 protein coding genes. Genome wide significance was defined as a *p*-value of 0.05/18,644 = 2.682 × 10^−6^.

### 2.9. Expression Analysis using SysFACE

Evaluation of expression of a given candidate gene and its neighboring genes was performed using the bioinformatics tool SysFACE (systems tool for craniofacial expression-based gene discovery; https://bioinformatics.udel.edu/research/sysface). For each gene, expression data for various time-points of murine embryonic development in organs specific for the phenotype under study (maxilla, frontonasal, palate) were identified from microarray-based genome-level gene expression profiles across various mouse embryonic orofacial tissues.

## 3. Results

### 3.1. SNV-Based Analysis 

A total of 615,168 autosomal markers passed QC for both samples. The genomic inflation factor lambda was 1.044. The Q–Q plot is shown in Figure S2.

In the imputed GWAS data, 228 SNVs at a total of 25 different loci yielded *p*-values < 10^−5^ and an INFO score > 0.8 (Table 1, Table S1, Figure S3). 

Genome-wide significance (*p* < 5 × 10^−8^) was reached by 63 SNVs at two loci. One of the genome-wide significant SNVs was rs73145631 at 12q23.1, a locus that has not been reported as an nsCL/P risk locus previously. It was subjected to a replication step in three independent case/control samples (Table 2). In none of the three replication samples, nor after combining the replication data in a meta-analysis was the association for this SNV replicated. The other 62 genome-wide significant SNVs were located at an already well-known nsCL/P susceptibility locus at chromosome 8q24.21. This locus was initially identified in the Central European case/control sample that served as a replication sample for the present study [8]. It was later replicated in many other samples—among others, the Mexican and the Yemeni replication samples that were used for replication in the present study [26,27]. This locus was therefore not included in the replication step of the present study.

This locus had not been reported as a recognized nsCL/P risk locus in previous studies. Therefore, rs56383345 was also subjected to replication in the three independent patient/control samples (Table 2). A nominally significant *p*-value was obtained for rs56383345 in both the European and the Yemeni sample (0.027 and 0.0099 respectively). The *p*-value obtained after combination of all three replication samples by meta-analysis was also nominally significant (*p* = 0.00167). However, no association signal was obtained in the Mexican sample only (*p* = 0.577). Rs56383345 is located in a 930 kb non-coding region. Evaluation of the 16p12.1 region with the bioinformatics tool SysFACE did not implicate any flanking genes as cleft candidate genes.

### 3.2. Replication of Previously Reported nsCL/P Susceptibility Loci 

Of the 40 previously reported nsCL/P susceptibility loci, 20 showed at least a nominally significant *p*-value (*p* < 0.05) in the present dataset (Table 3). For three of these loci, the lead SNV from the literature and the lead SNV in the present dataset were identical. At 15 of the 20 loci, a SNV in substantial linkage disequilibrium (LD) (*r^2^* > 0.6) with the lead SNV from the literature achieved a lower *p*-value. For two of the 20 loci, the lead SNVs were not in LD with the lead SNV from the literature. 

### 3.3. Gene-Based Evaluation and Gene-Set Analysis

**Figure 2:**
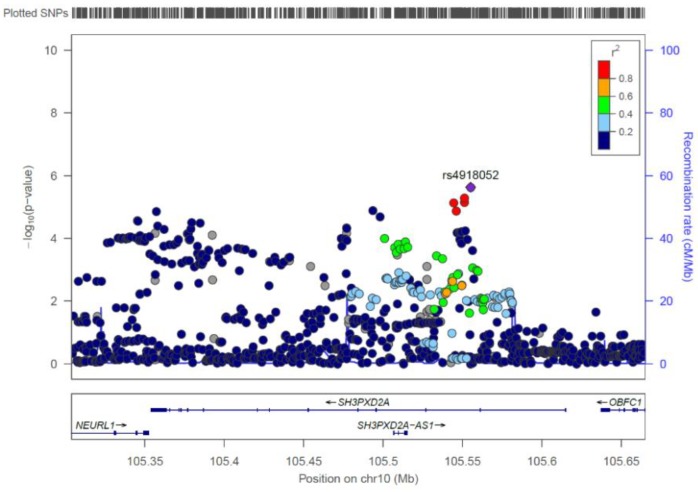
Regional association plot for the candidate gene *SH3PXD2A*.

**Figure 3:**
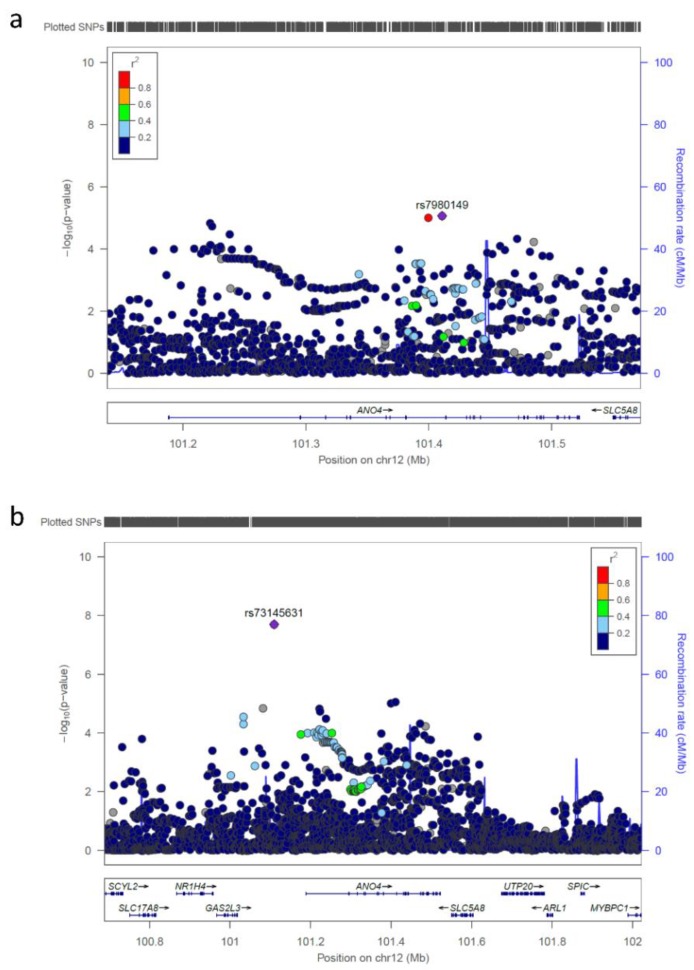
An unreported suggestive locus at chromosome 12q23.1 (**a**) Regional association plot showing a genome-wide significant marker upstream of the *anoctamin 4* (*ANO4*) gene. It is of note that in our dataset, there were no SNVs in LD with the lead SNV rs73145631. (**b**) Regional association plot for the *ANO4* gene.

For the gene-based evaluation, genome-wide significance was set by FUMAGWAS at *p* < 2.672 × 10^−6^ and was achieved by two different genes: (i) *SH3 And PX Domains 2A* (*SH3PXD2A*) at chromosome 10q24.33 (*p* = 1.82 × 10^−8^); and (ii) *anoctamin 4* (*ANO4*) at chromosome 12q23.1 (*p* = 1.46×10^−7^) (Figure 2, Figure 3, Figure S4, Table S2). To date, neither of these genes has been proposed as a candidate gene for clefting in humans. For *ANO4,* the SysFACE evaluation revealed no expression at any relevant embryonic time-points in tissues of relevance to lip and palate development in the mouse model (Figure S5). However, *Growth Arrest Specific 2 Like 3* (*GAS2L3*), which is located upstream of *ANO4*, is expressed in the murine maxilla at E11.5 to E12.5, and *Insulin-Like Growth Factor 1* (*IGF1*), which is located downstream, is expressed in the murine maxilla at E11.5 to E12.5 and also in the murine palate at E14.5. 

Genes with a suggestive *p*-value (2.672 × 10^−6^ < *p* < 10^−4^) in the gene analysis included the gene *Paired Box 7* (*PAX7*) at chromosome 1p36.13. *PAX7* is a well-known cleft candidate gene (Table 4). The *PAX7* locus has shown genome-wide association in two previous studies, and rare, potentially pathogenic variants located in or near *PAX7* have been reported in patients with orofacial clefting [30,31,32,33].

The gene-set analysis implemented in FUMAGWAS revealed twelve gene ontology (GO) gene sets with an adjusted *p*-value < 0.05 (Table S2). The two gene sets with the lowest *p*-values were GO_TISSUE_MORPHOGENESIS (adjusted *p* = 0.0259) and GO_KERATINOCYTE_PROLIFERATION (adjusted *p* = 0.0259). 

## 4. Discussion

The present report describes a GWAS performed in nsCL/P patients from the Netherlands and Belgium and unaffected controls from the Netherlands. The sample comprised 259 patients and 1187 controls, and thus represents a medium-sized nsCL/P cohort. Nonetheless, the replication of 20 of the 40 previously reported nsCL/P susceptibility loci—four of which were originally identified in Asian samples—demonstrated the power of the sample. Notably, at the well-established nsCL/P susceptibility locus at chromosome 8q24.21, the analyses identified 62 genome-wide significant SNVs. Among others, the results of the gene-set analysis prioritized GO_TISSUE_MORPHOGENESIS and GO_KERATINOCYTE_PROLIFERATION. This was consistent with the hypothesis that nsCL/P arises from the disturbed proliferation, adhesion, and apoptosis of cells in the facial prominences during embryogenesis, and demonstrated the validity of the present Dutch/Belgian sample [33].

The SNV-based analysis identified a novel locus at chromosome 16p12.1 yielding suggestive evidence of association (lead SNV rs56383345 with a *p* = 4.17 × 10^−7^). This association was replicated in two of the three replication series (Central European and Yemeni). The association was not replicated in the Mexican patient/control series. However, the MAF for this SNV in the Mexican series was <2%, so the sample would not be expected to have much power to detect a true association. The SNV rs56383345 maps to a 930 kb non-coding region at 16p12.1, and is not located in any currently known regulatory element [34]. The nearest flanking genes are *heparan sulfate-glucosamine 3-sulfotransferase 4* (*HS3ST4)* upstream and *C16orf82* downstream. Neither of these genes, nor any other genes near rs56383345, has any reported role in cleft development. Furthermore, no orofacial clefting has been reported in those patients from the DECIPHER database [35] who have a copy number variant encompassing the new suggestive susceptibility locus or either one of the flanking genes. 

Twenty of the 40 recognized nsCL/P susceptibility loci were replicated with at least a nominally significant *p*-value (*p* < 0.05) in the present study. At 3 of these 20 replicated loci, the lead SNV reported in the literature and the lead SNV in the present dataset were identical, and at 15 loci a SNV in LD (r^2^ > 0.6) with the lead SNV from the literature achieved a smaller *p*-value. Notably, at two of the 20 replicated loci, namely 1p36 and 3q29, additional peaks with lead SNVs not in LD with the lead SNV from the original literature were identified. This suggested that the original sample and the Dutch/Belgian sample have differing haplotype structures. 

In addition to the “typical” single-SNV analyses, the present study involved a gene analysis, which generated several interesting findings. In a gene analysis, genetic marker data are aggregated to the level of whole genes to test the joint associations of all markers in the gene with the phenotype [36]. Previous authors have suggested that this approach represents a potentially more powerful alternative to single-SNV analyses. The gene analysis approach has the advantage of considerably reducing the required number of tests, and renders possible detection of effects consisting of multiple weaker associations, which would otherwise be overlooked.

The present gene analysis prioritized *SH3PXD2A* at chromosome 10q24.33 as a candidate gene for nsCL/P. *SH3PXD2A* is a protein-coding gene with 15 exons and a size of 267 kb. The *SH3PXD2A* gene product is necessary for the formation and function of podosomes, which are structures located on the cellular surface that establish close contact with the extracellular matrix, and are involved in cell migration and matrix degradation [37]. Observations in zebrafish and knockout mice suggest that *SH3PXD2A* is also a potential risk gene for orofacial clefting [38,39]. In mammals, the primary and secondary palate forms from cranial-neural-crest-cell-derived mesenchymal protuberances, and any alteration in the growth, proliferation, movement, adhesion, or death of cells composing the palatal structures can affect palatal architecture and lead to orofacial clefting. *SH3PXD2A* encodes TKS5, a scaffold protein shown to be fundamental to zebrafish neural crest cell migration in vivo [38]. However, even stronger support for that *SH3PXD2A* may be a cleft candidate gene has been generated by Cejudo-Martin et al., who showed that disruption of the mouse *Sh3pxd2a* gene was associated with complete cleft of the secondary palate in 50–90% of mutant mice [39]. Of note, the fact, that the mouse model in Cejudo-Martin et al. has a cleft of the secondary palate but not a cleft lip, does not speak against this gene being also involved in nsCL/P formation in humans. The present gene analysis provides the first strong support for the involvement of *SH3PXD2A* in non-syndromic cleft formation in humans.

Gene evaluation of the present GWAS data also prioritized the gene *anoctamin 4* (*ANO4*) at chromosomal band 12q23.1. Interestingly one SNV, rs73145631, located 79 kb upstream of the transcription start site of this gene, achieved genome-wide significance in this dataset. The SNV association signal should be considered independent of the MAGMA gene analysis, which only took into account signals located between the transcription start and stop sites of the gene. The protein product of the candidate gene, ANO4, is a transmembrane protein from the anoctamin family. This protein family plays a key role in diverse physiological functions, including ion transport, phospholipid scrambling, and the regulation of other ion channels. While the ANO1 and ANO2 proteins have been functionally characterized, the roles of other family members, such as ANO4, remain poorly understood and controversial [40]. There is currently no convincing support for *ANO4* as a cleft susceptibility gene in the literature or publically available databases on gene expression, such as SysFace. However, it is possible that rather than being attributable to the *ANO4* gene itself, this association signal was generated by a regulatory element located intronically in *ANO4*, which influences the activity of another nearby gene. Notably, two genes in the vicinity of the *ANO4* signal are expressed at relevant embryonic time-points in tissues of relevance to lip and palate development in the mouse model: (i) *Growth Arrest Specific 2 Like 3* (*GAS2L3*), located upstream from *ANO4;* and (ii) *Insulin Like Growth Factor 1* (*IGF1*), located downstream [34]. In the present GWAS, the genome-wide significant association signal for rs73145631 in the *ANO4* upstream region could be interpreted as additional independent support for the *ANO4* locus, despite being based on the same data. However, for several reasons, these results must be interpreted with caution. First, no supportive association signal for rs73145631 was found in any of the three replication cohorts. Second, interpretation of the GWAS association signal for rs73145631 was hampered by the fact that no SNVs were in LD with this SNV in this dataset. Even though it was not replicated in any of the three replication samples, the finding may be a true association specific to the present Dutch/Belgian sample, or may represent a false positive signal from a single marker.

After Asians, Europeans represent the second most common ethnicity in published nsCL/P association studies. The largest ethnically homogenous GWAS sample to date included 2033 patients of Chinese ancestry [17]. The largest European SNV data set explored to date combined 1158 nsCL/P patients from two large studies in a genome-wide meta-analysis [20]. Compared to such studies, the present sample size was relatively small, but this sample was valid and allowed the generation of interesting results warranting further investigation. The present sample is also of value in terms of future meta-analyses of GWAS data. These meta-analyses will increase statistical power for locus discovery, and facilitate the elucidation of the genetic background of orofacial clefting. 

## 5. Conclusions

In summary, the present GWAS generated novel insights into the etiology of nonsyndromic orofacial clefting. The gene-based analysis provided strong support for *SH3PXD2A* as a candidate gene identified in animal models, and the SNV analysis identified two novel suggestive risk loci. The present results demonstrate how even medium-sized, clinically well characterized GWAS samples can improve knowledge of the genetic basis of nsCL/P.

## Figures and Tables

**Figure 1:**
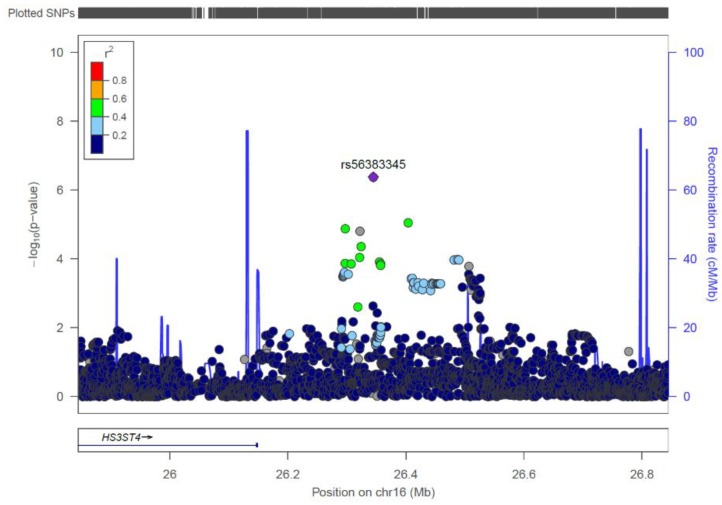
Regional association plot for a novel suggestive non-syndromic cleft lip with or without cleft palate (nsCL/P) locus on chromosome 16p12.1. Plotted SNVs include both genotyped and imputed variants.

**Table 1:** Twenty-five nsCL/P risk loci achieving genome-wide significant or suggestive evidence for association in imputed Dutch/Belgian genome-wide association study (GWAS).

LeadSNV_ID ^1^	Chromosome	Pos (hg19)	Alleles ^2^	Case Frequency A	Control Frequency A	All OR	*p*-Value ^3^
rs36068947	1	19003293	G/GC	0.592	0.702	1.62	1.06 × 10^-6^
rs11247713	1	28230494	A/G	0.165	0.261	1.78	2.54 × 10^-6^
rs12404189	1	81851687	T/C	0.951	0.899	0.46	4.48 × 10^-6^
rs145794647	1	213880572	A/G	0.915	0.964	2.46	3.17 × 10^-6^
rs1266381	1	236681990	A/**G**	0.68	0.781	1.68	1.49 × 10^-6^
rs13429389	2	2564600	G/A	0.713	0.807	1.68	6.34 × 10^-6^
rs1431903	2	168468315	T/C	0.113	0.192	1.86	8.76 × 10^-6^
rs112762347	5	13103319	G/A	0.952	0.986	3.56	5.80 × 10^-6^
rs4868099	5	170982499	T/C	0.156	0.089	0.53	4.29 × 10^-6^
rs141109174	7	2926872	G/GA	0.924	0.856	0.49	3.95 × 10^-6^
rs987525	8	129946154	C/A	0.61	0.748	1.89	8.73 × 10^-11^
rs1535462	10	102973872	A/G	0.623	0.518	0.65	5.69 × 10^-6^
rs4918052	10	105555131	G/A	0.303	0.203	0.58	2.34 × 10^-6^
rs17770307	10	115259535	G/C	0.984	0.942	0.26	2.59 × 10^-6^
rs148248623	12	23265077	CA/C	0.907	0.961	2.52	1.38 × 10^-6^
rs7980090	12	67951884	C/A	0.908	0.958	2.28	2.35 × 10^-6^
rs73145631	12	101109530	G/A	0.985	0.936	0.23	1.99 × 10^-8^
rs56814511	12	125789014	C/T	0.608	0.705	1.55	9.01 × 10^-6^
rs184467	13	29622636	**G**/T	0.428	0.316	0.62	1.53 × 10^-6^
rs10520788	15	96126414	T/C	0.973	0.924	0.34	6.19 × 10^-6^
rs56383345	16	26344915	G/C	0.922	0.845	0.46	4.17 × 10^-7^
rs11640952	16	78093932	G/T	0.693	0.587	0.63	6.22 × 10^-6^
rs7215555	17	29564603	G/A	0.231	0.331	1.64	3.28 × 10^-6^
rs61296704	17	75721588	G/A	0.932	0.972	2.55	8.22 × 10^-6^
rs73512449	19	18123050	G/C	0.888	0.946	2.19	7.00 × 10^-6^

^1^**underlined** = genome-wide significant SNVs, ^2^ major allele first, risk allele underlined, ^3^ only markers with a *p*-value < 10^−5^ are presented. Pos = position, OR = odds ratio. The 25 loci also included an interesting locus at chromosome 16p12.1. This locus gave a suggestive *p*-value (5 × 10^−8^ < *p* < 10^−5^), and its lead SNV was rs56383345, with a *p*-value of 4.17 × 10^−7^ (Figure 1).

**Table 2:** Replication of two interesting SNVs in three replication samples of different biogeographical backgrounds.

SNV-ID			*p*-Values after Genotyping of SNV in Respective Replication Sample				
SNV-ID	Chr.	Pos. (hg19)	Bonn ^1^	Mexico ^2^	Yemen ^3^	All ^4^	OR (95% CI)
rs73145631	12q23.1	101109530	0.544	n.a.*	0.714	0.488	1.18 (0.74–1.90)
rs56383345	16p12.1	26344915	**0.027**	0.577 **	**0.0099**	**0.00167**	1.53 (1.17–1.98)

**Bold** = nominal significant result, *italics* = risk allele in this subsample not identical with risk allele in Dutch/Belgian discovery sample, Chr. = chromosome, pos. = position, OR = odds ratio, CI = confidence interval, n.a. = not applicable. * MAF below 1% in controls, therefore excluded (notable: rs17447439: 0.9% in controls vs. 1.7% in cases), ** 1.7% in cases and 1.2% in controls. ^1^ nsCL/P case control sample of 223 nsCL/P patients and 978 controls of Central European descent (no overlap with Bonn GWAS). ^2^ 156 nsCL/P patients and 337 controls from the Chiapas, Mexico. ^3^ 231 nsCL/P patients and 422 controls from Yemen. ^4^combined analysis of Bonn, Mexico, and Yemen.

**Table 3:** *P*-values for 40 literature nsCL/P risk loci in imputed Dutch/Belgian GWAS.

Literature Risk Locus ^1^		Literature Lead SNV at Respective Locus				Better Dutch/Belgian GWAS lead SNV at Respective Locus			
Locus	Original Study	SNV	Pos. (hg19)	*p*-Value ^2^	OR	SNV ^3^	Pos (hg19)	*p*-Value ^2,3^	OR
**1p36**	Ludwig et al. 2012 [12]	rs742071	18979874	**2.11 × 10^-5^**	1.54	*rs36068947*	*19003293*	***1.06272* × 10^-6^**	*1.62*
1p22	Beaty et al. 2010 [11]	rs560426	94553438	0.299446	0.90	rs952499	94558425	0.0616217	0.83
**1q32.1**	Rahimov et al. 2008 [3]	rs642961	209989270	**0.00055997**	0.69	-	-	-	-
2p25.1	Yu et al. 2017 [17]	rs287982	9972442	0.898877	1.00	-	-	-	-
**2p24.2**	Leslie et al. 2016 [16]	rs7552	16733928	**4.84 × 10^-5^**	1.52	rs62122693	16734878	**3.10168 × 10^-5^**	1.56
**2p21_PKDCC_**	Ludwig et al. 2017 [19]	rs6740960	42181679	**0.00745325**	1.28	*rs17029056*	*42158304*	***0.00011259***	*1.52*
2p21_THADA_	Ludwig et al. 2012 [12]	rs7590268	43540125	0.13198	1.20	rs6544652	43626212	0.0881938	1.23
**3p11.1**	Ludwig et al. 2012 [12]	rs7632427	89534377	**0.0342897**	0.81	rs3792572	89456555	**0.0101378**	0.77
**3q12.1**	Beaty et al. 2013 [13]	rs793464	99626028	**8.21 × 10^-5^**	0.70	rs9832134	99836722	**4.67043 × 10^-5^**	0.66
**3q28**	Leslie et al. 2017 [20]	rs76479869	189553372	**0.00247675**	1.77	rs17447439	189549423	**5.67543 × 10^-5^**	2.26
**3q29**	Mostowska et al. 2018 [18]	rs338217	197026927	0.0986085	0.85	rs34099552	196799735	**0.0228988**	1.27
4p16.2	Yu et al. 2017 [17]	rs1907989	4818925	0.751776	1.05	rs10937893	4810491	0.649752	0.94
4q28.1	Yu et al. 2017 [17]	rs908822	124906257	0.657599	0.89	rs76837304	124868111	0.532867	0.86
5p12	Yu et al. 2017 [17]	rs10462065	44068846	0.258344	1.18	rs139738798	44183419	0.109377	1.26
8p11.23	Yu et al. 2017 [17]	rs13317	38269514	0.774445	0.97	rs75168396	38014429	0.3038	1.11
**8q21**	Ludwig et al. 2012 [12]	rs12543318	88868340	**0.00118317**	0.73	-	-	-	-
8q22.1	Yu et al. 2017 [17]	rs957448	95541302	0.560023	0.94	rs4442106	95609488	0.0626976	0.83
**8q24**	Birnbaum et al. 2009 [8]	rs987525	129946154	**8.73 × 10^-11^**	1.89	-	-	-	-
**9q22.2**	Yu et al. 2017 [17]	rs10908902	92224825	**0.0178356**	1.31	rs2031970	92204172	**0.00252225**	1.41
9q22.32	Yu et al. 2017 [17]	rs10512248	98259703	0.0938744	0.84	rs28591501	98278644	0.0696448	0.82
**9q21.33**	Moreno et al. 2009 [4]	rs3758249	100614140	**0.00519618**	1.32	rs7033765	100591705	**0.00127545**	1.38
**10q25**	Mangold et al. 2010 [10]	rs7078160	118827560	**0.00019291**	1.60	rs5788208	118836076	**0.000106433**	1.63
12q13.13	Yu et al. 2017 [17]	rs3741442	53346750	0.105465	0.31	-^4^	-^4^	-^4^	-^4^
**12q13.2**	Yu et al. 2017 [17]	rs705704	56435412	**0.0418832**	1.22	rs773107	56369506	**0.0210978**	1.26
12q21.1	Yu et al. 2017 [17]	rs2304269	72080272	0.483316	0.86	rs11178895	72089411	0.321804	0.84
**13q31.1**	Ludwig et al. 2012 [12]	rs8001641	80692811	**0.00268945**	1.34	rs11841646	80679302	**0.00135074**	1.37
14q22.1	Ludwig et al. 2017 [19]	rs4901118	51856109	0.367607	1.10	rs60454187	51856566	0.279705	0.9
14q22.1	Yu et al. 2017 [17]	rs7148069	51839645	0.731853	1.03	-	-	-	-
14q32.13	Yu et al. 2017 [17]	rs1243572	95379499	0.343413	1.12	rs1243561	95369886	0.201146	1.16
**15q13**	Ludwig et al. 2016 [15]	rs1258763	33050423	0.0521438	1.19	rs13329310	33052553	**0.0499003**	0.83
15q22.2	Ludwig et al. 2012 [12]	rs1873147	63312632	0.469881	1.08	rs12902152	63313968	0.167601	1.19
15q24	Ludwig et al. 2017 [19]	rs28689146	75005575	0.442303	1.08	-	-	-	-
**16p13.3**	Sun et al. 2015 [14]	rs8049367	3980445	0.159099	1.13	rs11076792	3968567	**0.00316807**	1.33
17q13.1	Beaty et al. 2010 [11]	rs9891446	8935416	0.186388	1.14	-	-	-	-
**17q21.32**	Yu et al. 2017 [17]	rs1838105	45008935	**0.00231174**	0.73	rs197907	44982081	**0.000282387**	1.45
17q22	Mangold et al. 2010 [10]	rs227727	54776955	0.180057	1.14	-	-	-	-
**17q23.2**	Leslie et al. 2016 [16]	rs1588366	61076428	**0.00901131**	0.75	rs72843145	61052949	**0.0057287**	0.72
19p13.3	Ludwig et al. 2017 [19]	rs3746101	2050823	0.929281	0.95	-	-	-	-
19q12	Leslie et al. 2016 [16]	rs73039426	33520961	0.26959	1.20	-	-	-	-
**20q12**	Beaty et al. 2010 [11]	rs13041247	39269074	**0.0101081**	0.77	rs34753522	39278391	**0.00037345**	0.69

pos. = position, OR = odds ratio, - = no SNV with smaller *p*-value at this locus compared to literature lead SNV. ^1^ **bold** if locus reached a nominal significant *p*-value in Dutch/Belgian GWAS. ^2^ *p*-value in **boldface** if nominal significant. ^3^normal letters if in LD with literature lead SNV (r2 > 0.6; r2 taken from LD Link), *italics* if not in LD with literature lead SNV and *p* < 10^−4^. ^4^ MAF of lead SNV in Dutch/Belgian GWAS below 0.01, did not pass QC.

**Table 4:** Gene analysis with MAGMA as implemented in FUMAGWASGene ^1.^

	Chromosome	*p*-Value ^1^
***SH3PXD2A***	10	**2.3729 × 10^−8^**
***ANO4***	12	**1.4588 × 10^−7^**
*CMSS1*	3	2.9078 × 10^−6^
*FILIP1L*	3	7.1274 × 10^−6^
*CLEC3A*	16	1.0871 × 10^−5^
*PAX7^2^*	1	1.9157 × 10^−5^
*CCDC140*	2	4.4909 × 10^−5^
*ESR1*	6	7.1554 × 10^−5^
*IFITM3*	11	8.5273 × 10^−5^
*PANX1*	11	8.7342 × 10^−5^
*BLMH*	17	9.5824 × 10^−5^

^1^**bold** = genome wide significant genes/*p*-values. ^2^ previously known nsCL/P susceptibility gene.

## Supplementary Materials

The following are available online at https://www.mdpi.com/2073-4425/10/12/1023/s1, Figure S1: Multidimensional Scaling (MDS) analysis, Dutch/Belgian GWAS individuals in blue, patients open diamonds, controls dots; Figure S2: QQ-plot; Figure S3: Manhattan plot of summary statistics; Figure S4: Manhattan plot of gene-based test; Figure S5: UCSC tracks of ANO4 region including SysFace data; Table S1–S2: (S1) 25 nsCL/P risk loci with genome-wide significant or suggestive association in imputed Dutch/Belgian GWAS, (S2) Gene-set analysis.
