# Supplementary material for: Non-Syndromic Cleft Lip with or without Cleft Palate: Genome-Wide Association Study in Europeans Identifies a Suggestive Risk Locus at 16p12.1 and Supports SH3PXD2A as a Clefting Susceptibility Gene
Source: Genes (Basel). 2019 Dec 7;10(12):1023. doi: 10.3390/genes10121023 (PMC6947597; doi:10.3390/genes10121023)
Supplement: Supplementary file 1 [file genes-10-01023-s001.zip › Suppl.Fig. 2_QQ-Plot_R1n.pdf]

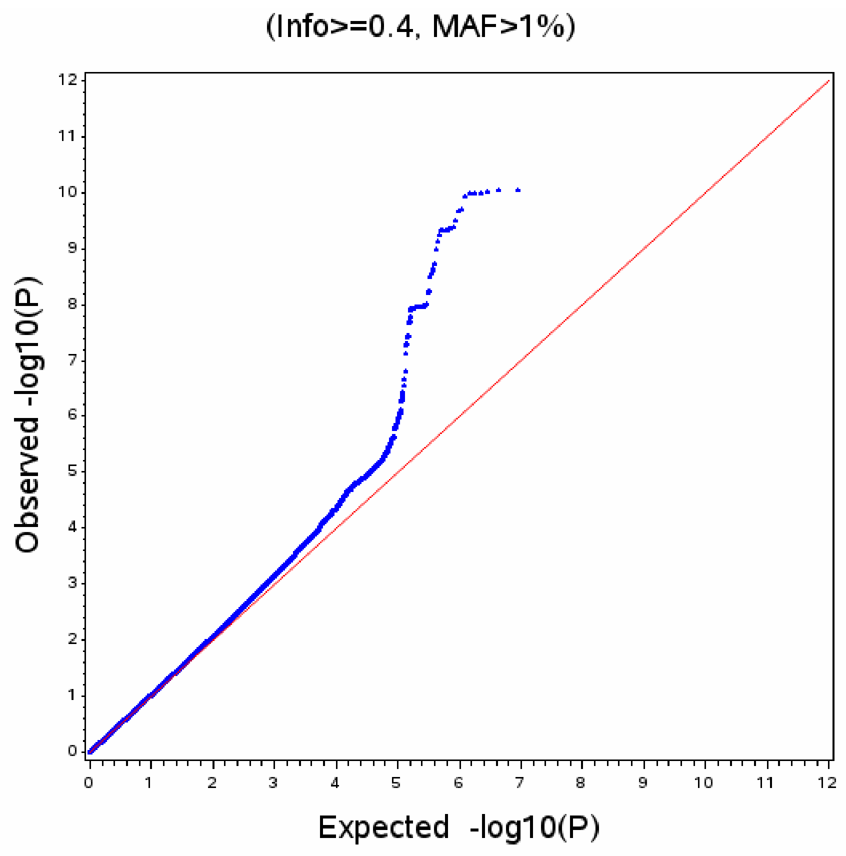

Supplementary Figure 2: QQ-plot demonstrating the number and magnitude of observed associations between SNPs and nsCL/P, compared to the association statistics expected under the null hypothesis of no association.
