# Supplementary material for: Non-Syndromic Cleft Lip with or without Cleft Palate: Genome-Wide Association Study in Europeans Identifies a Suggestive Risk Locus at 16p12.1 and Supports SH3PXD2A as a Clefting Susceptibility Gene
Source: Genes (Basel). 2019 Dec 7;10(12):1023. doi: 10.3390/genes10121023 (PMC6947597; doi:10.3390/genes10121023)
Supplement: Supplementary file 1 [file genes-10-01023-s001.zip › Suppl.Fig. 3_Manhattan Plot imputed GWAS_R1.pdf]

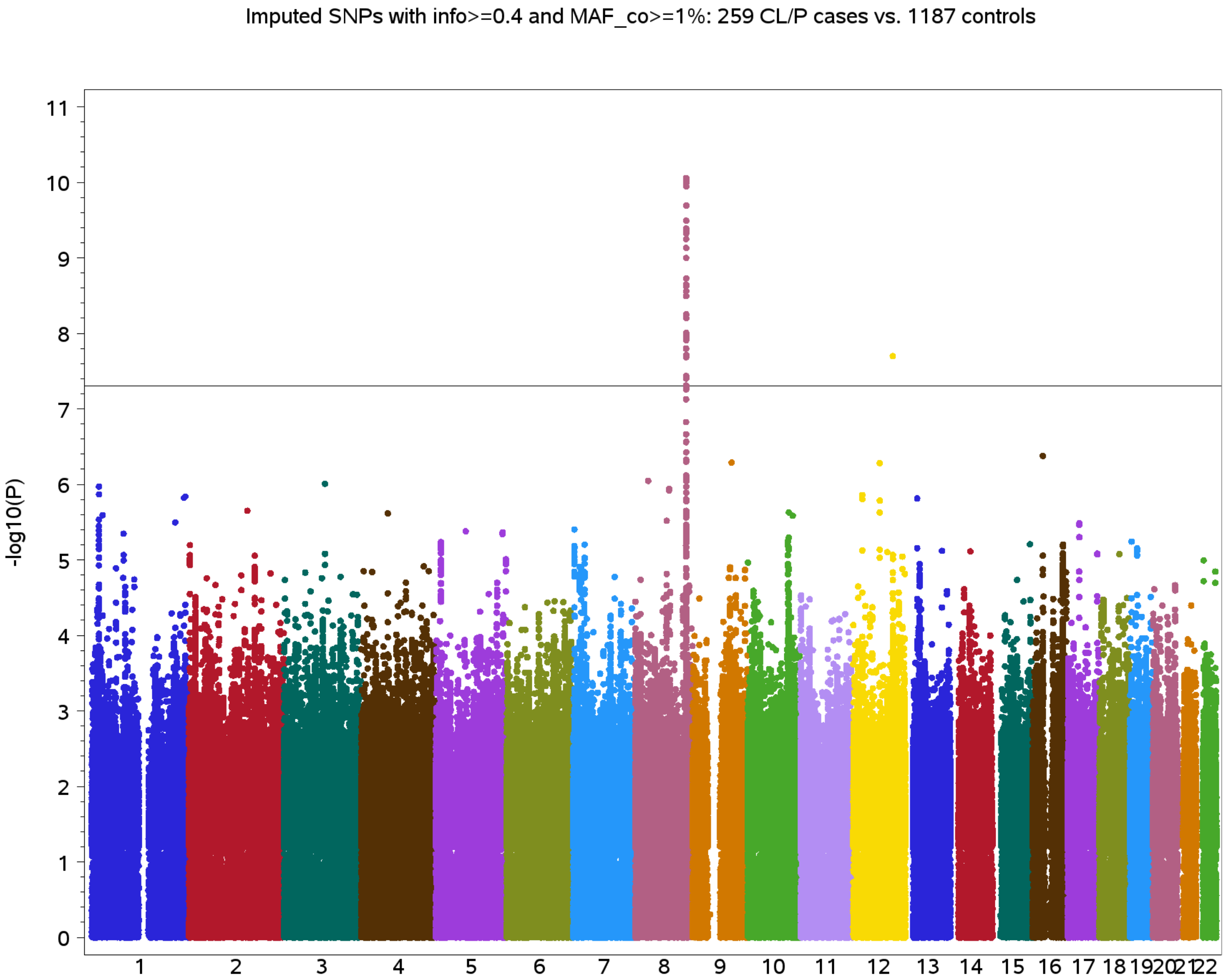

Supplementary Figure 3: Manhattan plot of summary statistics for nsCL/P. For each of the autosomal variants within the GWAS (INFO > 0.4 % and minor allele frequency in controls > 1%), -log10(p) association results are plotted along the chromosomal distribution. Grey line indicates genome-wide significance (p-value < 5 × 10E-08).
