# Supplementary material for: Non-Syndromic Cleft Lip with or without Cleft Palate: Genome-Wide Association Study in Europeans Identifies a Suggestive Risk Locus at 16p12.1 and Supports SH3PXD2A as a Clefting Susceptibility Gene
Source: Genes (Basel). 2019 Dec 7;10(12):1023. doi: 10.3390/genes10121023 (PMC6947597; doi:10.3390/genes10121023)
Supplement: Supplementary file 1 [file genes-10-01023-s001.zip › Suppl.Fig. 4_Manhatten Plot gene Analysis_R1n.pdf]

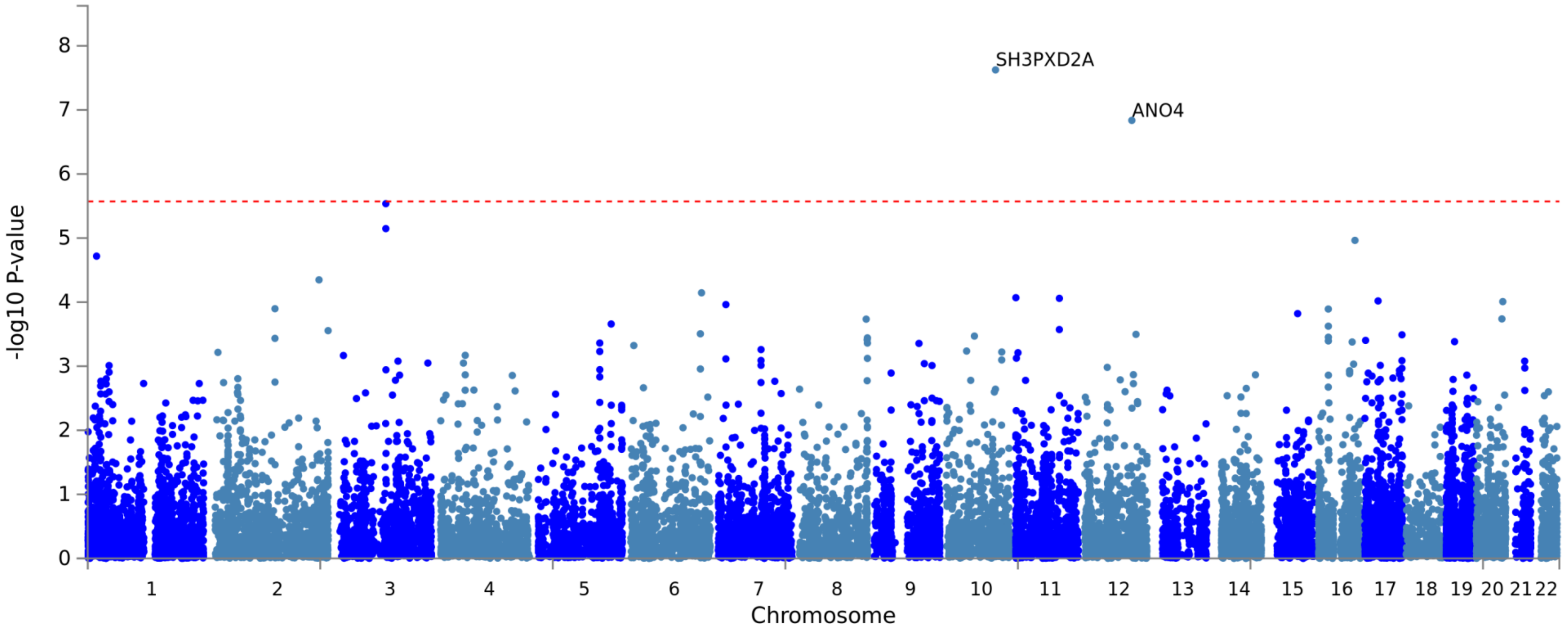

Supplementary Figure 6: Results of the gene analysis as exported from FUMA. Input SNVs (INFO ≥ 0.6 and minor allele frequency in controls > 1%), were mapped to 18,644 protein coding genes. For each of the autosomal genes association results are plotted along the chromosomal distribution. Red dotted line indicates genome-wide significance (p-value of 2.682×10E-06).
