# Supplementary material for: Non-Syndromic Cleft Lip with or without Cleft Palate: Genome-Wide Association Study in Europeans Identifies a Suggestive Risk Locus at 16p12.1 and Supports SH3PXD2A as a Clefting Susceptibility Gene
Source: Genes (Basel). 2019 Dec 7;10(12):1023. doi: 10.3390/genes10121023 (PMC6947597; doi:10.3390/genes10121023)
Supplement: Supplementary file 1 [file Suppl.Fig. 5_SysFace ANO4 region_R1.pdf › Suppl.Fig. 5_SysFace ANO4 region_R1.pdf]

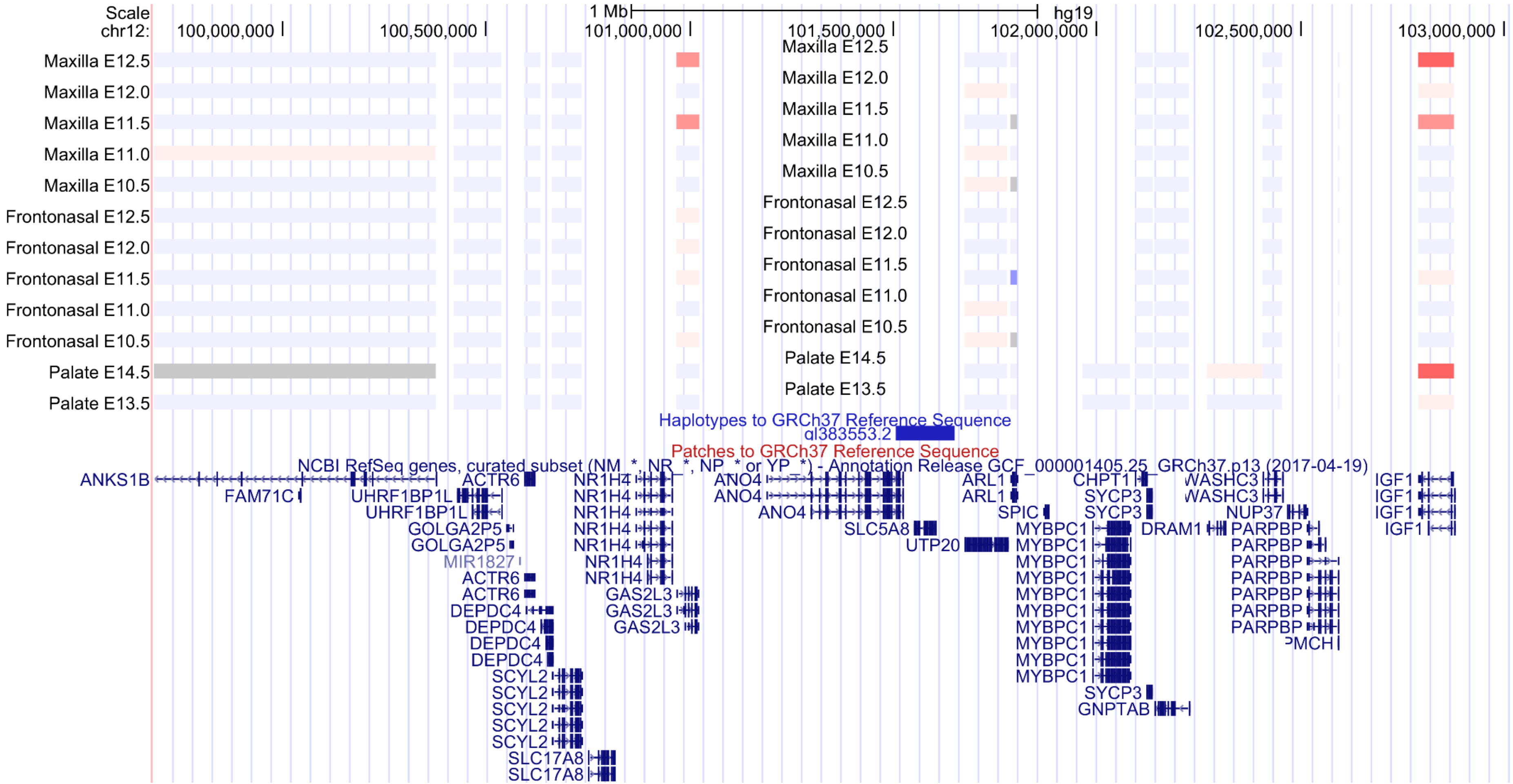

Supplementary Figure 4: SysFACE expression data for genes (RefSeq genes) located at 12p12.1 locus (*ANO4*) at various time points during human embryonic development (embryonic days E10.5-E14.5) and in anatomical structures relevant for the phenotype under study (Maxilla, Frontonasal, Palate). Red bars indicates high specific expression for two of the genes (*GAS2L3* and *IGF1*) in close proximity to *ANO4*.
